# TNF-α is a predictive marker in distinguishing myeloproliferative neoplasm and idiopathic erythrocytosis/thrombocytosis: development and validation of a non-invasive diagnostic model

**DOI:** 10.3389/fonc.2024.1369346

**Published:** 2024-03-22

**Authors:** Zhenhao Wang, Yu Mei, Zhuming Yang, Qiang Gao, Hao Xu, Zhiqiang Han, Zhenya Hong

**Affiliations:** ^1^ Department of Hematology, Tongji Hospital, Tongji Medical College, Huazhong University of Science and Technology, Wuhan, Hubei, China; ^2^ Department of Obstetrics and Gynecology, Tongji Hospital, Tongji Medical College, Huazhong University of Science and Technology, Wuhan, Hubei, China; ^3^ Cancer Biology Research Center (Key Laboratory of the Ministry of Education), Tongji Hospital, Tongji Medical College, Huazhong University of Science and Technology, Wuhan, Hubei, China

**Keywords:** myeloproliferative neoplasm, TNF-α, nomogram, diagnostic predictive model, cytokine

## Abstract

**Purpose:**

Philadelphia-chromosome negative myeloproliferative neoplasms (MPN) exhibit phenotypic similarities with JAK/STAT-unmutated idiopathic erythrocytosis and thrombocytosis (IE/IT). We aimed to develop a clinical diagnostic model to discern MPN and IE/IT.

**Methods:**

A retrospective study was performed on 77 MPN patients and 32 IE/IT patients in our center from January 2018 to December 2023. We investigated the role of hemogram, cytokine and spleen size in differentiating MPN and IE/IT among newly onset erythrocytosis and thrombocytosis patients. Independent influencing factors were integrated into a nomogram for individualized risk prediction. The calibration and discrimination ability of the model were evaluated by concordance index (C-index), calibration curve.

**Results:**

MPN had significantly higher TNF-α level than IE/IT, and the TNF-α level is correlated with MF-grade. Multivariable analyses revealed that TNF-α, PLT count, age, size of spleen were independent diagnostic factors in differentiating MPN and IE/IT. Nomograms integrated the above 4 factors for differentiating MPN and IE/IT was internally validated and had good performance, the C-index of the model is 0.979.

**Conclusion:**

The elevation of serum TNF-α in MPN patients is of diagnostic significance and is correlated with the severity of myelofibrosis. The nomogram incorporating TNF-α with age, PLT count and spleen size presents a noteworthy tool in the preliminary discrimination of MPN patients and those with idiopathic erythrocytosis or thrombocytosis. This highlights the potential of cytokines as biomarkers in hematologic disorders.

## Background

The classical Philadelphia-chromosome negative myeloproliferative neoplasms (MPN) represent a spectrum of clonal disorders originating from hematopoietic stem cells, wherein the neoplastic cells maintain their capability to differentiate into relative mature forms. MPN including four main subtypes: essential thrombocytosis (ET), polycythemia vera (PV), pre-fibrotic primary myelofibrosis (pre-PMF) and overt primary myelofibrosis (Overt-PMF) (1, 2). MPN patients typically share common genetic basis of acquired gain-of-function mutation of driver genes (JAK2, CALR and MPL), and exhibit overlapping or similar clinical features, such as peripheral cytosis, fatigue and night sweats; and hepatosplenomegaly could present in PV and PMF patients (3, 4). The phenotypes of different subtypes of MPN can be quite similar, and the phenotypes of MPN can also mimic that of other myeloid neoplasms and even benign hematologic disorders, especially JAK/STAT-unmutated idiopathic erythrocytosis or thrombocytosis (IE/IT) (5). Accurate and timely differentiation between MPN and IE/IT is pivotal for guiding appropriate clinical interventions. However, the diagnostic assessment of IE/IT remains challenging, as the diagnosis of IE/IT is typically a diagnosis of exclusion, wherein potential possibilities of MPN are mandatory to be ruled out, while erythrocytosis or thrombocytosis secondary to other underlying conditions should be excluded, for example, cardiopulmonary diseases, hepatic or renal tumors. Therefore, undergoing invasive bone marrow biopsy and driver gene mutation testing are mandatory for IE/IT patients to exclude the probability of MPN (6–9).

Aberrant proinflammatory signaling stands out as a defining feature of MPN, particularly evident in Overt-PMF, a series of interleukins (IL) and cytokines had been reported to be associated with pathogenesis of MPN, including IL-1β, IL-2R, IL-6, IL-8, IL-10, TNF-α, TGF-β and more, while CXCL4, CXCL8 and IL-4 were reported to associated with fibrotic progression (10–14). In addition, immune microenvironment plays an important role in the pathogenesis of MPN, myeloid-derived suppressor cells (MDSCs) and their secretion of pro-fibrotic cytokines also exert profound effect on the bone marrow microenvironment of MPN patients, impacting both the progression of fibrosis and patient survival through immune regulatory mechanisms and inflammation pathways (15, 16). Understanding the characteristics of cytokines is essential for better manage MPN patients, as well as contributing to the discovery of biomarkers for non-invasive auxiliary diagnostic methods for distinguishing MPN and IE/IT, alongside bone marrow biopsy.

In this retrospective study, we reported the clinical and laboratory characteristics in 77 MPN patients and 32 IE/IT patients, and we developed and validated a TNF-α incorporated nomogram (utilizing hemogram, TNF-α, age, and ultrasonography of the spleen) designed to discern MPN and IE/IT.

## Methods

### Study design

**Figure 1:**
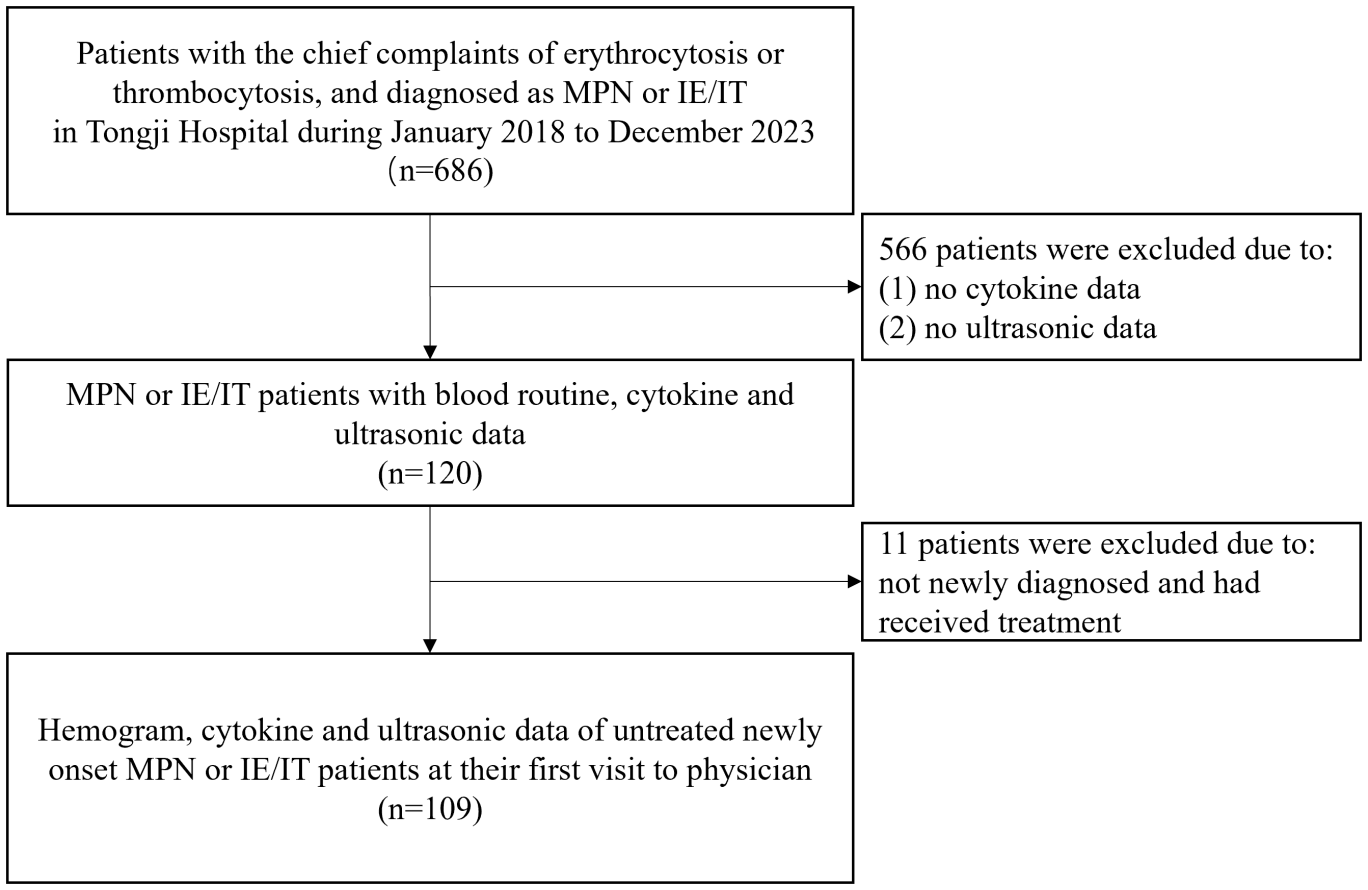
Flowchart of patient selection and data collection.

This study was a retrospective observational study, performed on 77 MPN patients and 32 IE/IT patients in our center from January 2018 to December 2023. All patients and their data were collected from the meta-data platform of Tongji Hospital, with patients’ name anonymized. The inclusion criteria were as follows: (1) diagnosed as ET, PV, Pre-PMF, Overt-PMF or IE/IT according to the International Consensus Classification of Myeloid Neoplasms and Acute Leukemias, confirmed by integration of bone marrow biopsy, genomic and clinical data (1). IE/IT were divided into idiopathic erythrocytosis (IE) and idiopathic thrombocytosis (IT), defined as having elevated hemoglobin (>165g/L for male and >160g/L for female) without an increase in platelets, with normal bone marrow biopsy and neither driver gene mutation nor other clonal markers; and having elevated platelets (>450×10^9^/L) without an increase in hemoglobin, with normal bone marrow biopsy and no gene mutation, respectively. Both IE and IT had no identified cause for their peripheral cytosis (2) had available data of blood routine, cytokine panel of IL-1β, IL-2R, IL-6, IL-8, IL-10, TNF-α, and spleen ultrasound examination at their first visit to physicians; (3) MPN patients had no additional concurrent diseases. The flowchart of enrollment was showed in **Figure 1**. All included patients underwent bone marrow biopsy and first-generation sequencing of MPN driver genes. Patients were diagnosed with MPN when their bone marrow biopsies support MPN, and driver gene mutations were detected by first generation sequencing; Patients were diagnosed with IE/IT only when bone marrow biopsy and first-generation sequencing did not support a diagnosis of MPN and there was unexplained erythrocytosis or thrombocytosis after excluding potential primary diseases.

The included variables were age, gender, white blood cell (WBC) count, hemoglobin (Hb) level, mean corpuscular volume (MCV), platelet (PLT) count, platelet distribution width (PDW), large platelet ratio (P-LCR), basophil, erythropoietin (EPO), lactate dehydrogenase (LDH), interleukin-1β (IL-1β), IL-2R, IL-6, IL-8, IL-10, tumor necrosis factor-α (TNF-α), size of spleen. The hemogram was obtained through blood routine test, LDH was obtained through lactate substrate method, IL-1β, IL-2R, IL-6, IL-8, IL-10, TNF-α and EPO were obtained through chemiluminescence assay of serum samples, the size of spleen was measured by ultrasound examination of spleen.

### Statistical analysis

Statistical analysis was conducted using R 4.2.1 (R Core Team, Vienna, Austria). Categorical variables were presented as numbers (%) and compared using The Chi-square test or Fisher’s exact test, as appropriate. Continuous variables were presented as median with interquartile range (IQR), and tested by rank sum test. The factors associated with diagnostic significance were assessed using multivariable logistic regression analysis. To choose the potential predictive features from selected patients, we utilized the least absolute shrinkage and selection operator (LASSO) regression. Nomograms were developed to evaluate the probability of MPN of patients. We used Harrell’s concordance-index (C-index) and the receiver-operating characteristic (ROC) curves with the calculated area under the curve (AUC) to assess the performances of the model. The calibration curve was used to evaluate the discrimination and calibration of the nomogram. Based on the nomogram, the points against each factor can be counted. The significance was assessed with two-sided p values less than 0.05. *OR* represented Odds ratio. *SE*: standard error.

## Results

### Hemogram and cytokine characteristics of MPN and IE/IT patients

A total of 109 untreated myeloproliferative neoplasm (MPN) or idiopathic erythrocytosis/thrombocytosis (IE/IT) patients who underwent hemogram and cytokine test at their first visit to physician were enrolled in our study. **Table 1** displayed the demographic characteristics of these patients. The median age of all patients was 53 years, IE and IT patients having the youngest median age of 46 and 27 years, respectively. The median age of all MPN subtypes almost exceeded 50 years. Median WBC count of all patients was 8.5×10^9^/L, Overt-PMF and PV patients had highest count of 14.1 and 13.2×10^9^/L, respectively, while median count of other patients were within normal range (<10.0×10^9^/L). Median Hb levels were 185g/L for IE and 178g/L for PV patients, while PMF patients exhibited lowest median Hb levels, both Pre-PMF and Overt-PMF had median Hb levels below 120g/L, IT and ET patients had moderate Hb level. Notably, IE patients had highest median MCV of 93fL. Median PLT count of all patients was 583×10^9^/L, IE patients had lowest median count of 193×10^9^/L, followed by Overt-PMF with 348×10^9^/L, IT and PV patients had median count of 528 and 577×10^9^/L respectively, ET patients had highest median PLT count of 821×10^9^/L. Overt-PMF and IE patients had higher median PDW (14.8 fL and 13.1 fL) and median P-LCR (35% and 31%), IT patients had a lower median PDW of 10.15fL and median P-LCR of 20%. As for Basophil, Overt-PMF patients had highest median basophil count of 0.14×10^9^/L, followed by Pre-PMF with 0.09×10^9^/L, then PV and ET both had median basophil count of 0.05×10^9^/L, IE and IT had lowest median basophil count of 0.02×10^9^/L. Median LDH level of IE and IT were both within normal range(<214U/L), gradually increasing in ET, PV, Pre-PMF and Overt-PMF, with Overt-PMF reaching over twice the normal upper limit. Median EPO level was lowest in PV patients (2pg/ml), while median EPO level of IE patients was 10pg/ml, Overt-PMF patients had highest median EPO level of 34pg/ml. Regarding spleen size, Overt-PMF was largest with median of 6.3cm, followed by PV (4.1cm), Pre-PMF (4.0cm) and ET (3.8cm), IE and IT had smallest spleen, with median size of 3.6cm and 3.2cm.

**Table 1:** Hemogram and cytokine profiles of included patients.

Characteristic	Diagnosis							p-value^2^
	Overall, N = 109^1^	IE, N = 18^1^	IT, N = 14^1^	ET, N = 29^1^	PV, N = 19^1^	Pre-PMF, N = 13^1^	Overt-PMF, N = 16^1^	
**Age, yrs**	53 (39, 65)	46 (29, 55)	27 (21, 45)	48 (36, 64)	59 (52, 68)	57 (47, 77)	63 (56, 67)	<0.001
**Gender**								<0.001
Male	55 (50%)	18 (100%)	1 (7%)	18 (62%)	5 (26%)	4 (31%)	9 (56%)	
Female	54 (50%)	0 (0%)	13 (93%)	11 (38%)	14 (74%)	9 (69%)	7 (44%)	
**WBC, ×10^9/L**	8.5 (7.0, 13.9)	7.1 (6.2, 7.8)	9.4 (7.2, 11.2)	8.4 (7.1, 10.4)	13.2 (8.4, 20.6)	10.0 (8.1, 16.3)	14.1 (7.7, 17.6)	<0.001
**Hb, g/L**	139 (115, 170)	185 (182, 189)	126 (116, 134)	133 (115, 143)	178 (165, 194)	118 (93, 139)	107 (74, 118)	<0.001
**MCV, fL**	89 (84, 92)	93 (88, 95)	85 (83, 86)	89 (87, 92)	86 (83, 91)	90 (86, 92)	88 (81, 95)	0.021
**PLT, ×10^9/L**	583 (294, 888)	193 (154, 242)	528 (474, 615)	821 (692, 1,099)	577 (419, 863)	932 (806, 1,198)	348 (159, 566)	<0.001
**PDW, fL**	11.90 (10.30, 13.90)	13.10 (11.00, 14.05)	10.15 (9.55, 11.98)	11.20 (9.80, 12.60)	12.80 (11.65, 14.90)	11.10 (10.60, 12.10)	14.80 (12.78, 16.53)	<0.001
**P-LCR, %**	27 (21, 32)	31 (25, 34)	20 (18, 28)	24 (19, 30)	29 (24, 36)	23 (22, 27)	35 (30, 39)	<0.001
**Basophil, ×10^9/L**	0.05 (0.02, 0.10)	0.02 (0.02, 0.03)	0.02 (0.01, 0.05)	0.05 (0.02, 0.11)	0.05 (0.04, 0.08)	0.09 (0.03, 0.16)	0.14 (0.07, 0.23)	<0.001
**LDH, U/L**	231 (187, 316)	187 (171, 225)	179 (146, 194)	231 (206, 271)	256 (219, 276)	316 (249, 394)	520 (334, 797)	<0.001
**EPO, pg/ml**	7 (3, 15)	10 (7, 15)	8 (7, 12)	6 (4, 18)	2 (2, 3)	10 (6, 15)	34 (7, 257)	<0.001
**IL-1b, pg/ml**	5 (5, 10)	5 (5, 9)	5 (5, 5)	5 (5, 5)	18 (6, 50)	5 (5, 7)	8 (5, 11)	<0.001
**IL-2R, pg/ml**	416 (298, 574)	327 (243, 351)	349 (267, 424)	456 (308, 562)	505 (292, 574)	430 (365, 542)	800 (480, 1,114)	<0.001
**IL-6, pg/ml**	4 (2, 9)	5 (2, 8)	3 (2, 6)	3 (2, 9)	5 (2, 9)	4 (3, 8)	7 (4, 15)	0.420
**IL-8, pg/ml**	22 (8, 86)	56 (7, 214)	9 (6, 22)	21 (10, 47)	26 (11, 51)	12 (8, 43)	42 (23, 184)	0.062
**IL-10, pg/ml**	5.00 (5.00, 5.00)	5.00 (5.00, 5.00)	5.00 (5.00, 5.00)	5.00 (5.00, 5.00)	5.00 (5.00, 6.85)	5.00 (5.00, 5.00)	5.00 (5.00, 5.00)	0.145
**TNF-a, pg/ml**	14 (9, 23)	7 (6, 12)	7 (7, 10)	13 (9, 17)	25 (18, 35)	18 (16, 22)	37 (23, 46)	<0.001
**Size of spleen, cm**	3.80 (3.30, 4.40)	3.55 (3.20, 3.90)	3.20 (3.20, 3.50)	3.80 (3.40, 4.10)	4.10 (3.65, 4.60)	4.00 (3.50, 4.30)	6.30 (4.70, 6.85)	<0.001

^1^Median (IQR); n (%).

^2^Kruskal-Wallis rank sum test; Pearson’s Chi-squared test.

**Figure 2:**
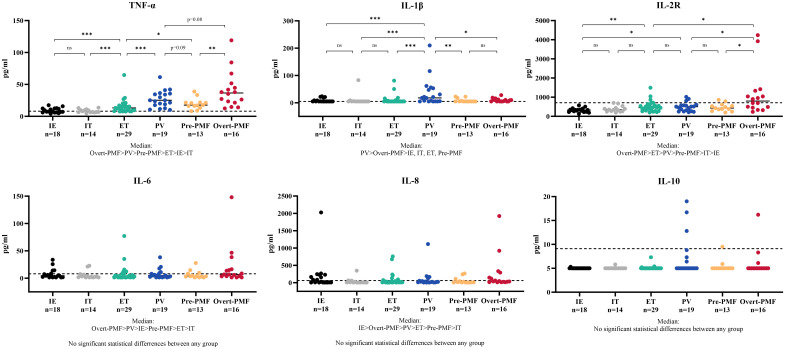
Cytokine Characteristics of MPN and IE/IT. MPN, myeloproliferative neoplasm; IE/IT, idiopathic erythrocytosis and idiopathic thrombocytosis. The “* ”represents P < 0.05, “**” represents P < 0.01, and “ ***” represents P < 0.001. ns, no significance.

The cytokine profile was showed in **Figure 2**. The disparity of TNF-α is most pronounced among the six cytokines, with Overt-PMF patients exhibiting the highest median level (37pg/ml), followed by PV (25pg/ml), Pre-PMF (18pg/ml) and ET (13pg/ml). Notably, each subtype of MPN demonstrated a significantly elevation of TNF-α compared to IE/IT (p<0.001). In addition, median IL-1β levels in PV (18pg/ml) patients are higher than in other subtypes of MPN or IE/IT (5pg/ml), followed by Overt-PMF (8pg/ml). IL-2R level was also consistently elevated in all subtypes of MPN compared to IE/IT (p<0.001), however, all median values remain within normal range (<710pg/ml) except for Overt-PMF (800pg/ml).

### Correlation of TNF-α with the severity of myelofibrosis

**Figure 3:**
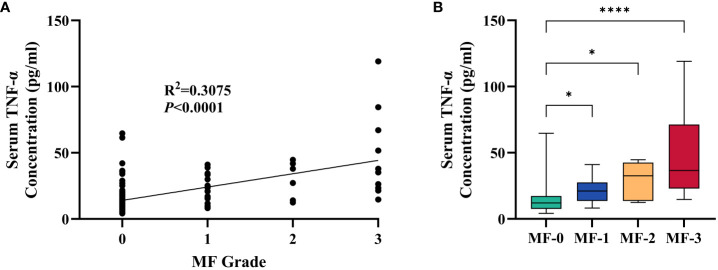
Correlation between Serum TNF-α Concentration and MF Grade of Myelofibrosis. **(A)** Correlation between serum TNF-α concentration and MF grade of myelofibrosis. **(B)** Serum TNF-α concentration of patients in different MF grade. The “* ”represents P < 0.05 and the “ ****” represents P < 0.0001.

It is noteworthy that there is a certain correlation between serum TNF-α and the grade of myelofibrosis in MPN and IE/IT patients. **Table 2** presents the MF grades of patients in each group. Serum TNF-α concentration showed a certain degree of correlation with MF grade (R^2^ = 0.3075, p<0.0001), and patients in MF-1 or higher MF grades exhibit significantly higher serum TNF-α levels compared to those in MF-0 (**Figures 3A, B**). Interestingly, although the proportion of patients in MF-1 among Pre-PMF patients is significantly higher than that among ET and PV patients (p=0.0004 and 0.029, respectively), the serum TNF-α levels in PV are higher than in Pre-PMF (p=0.09).

**Table 2:** MF grade of included patients.

MF-Grade	Diagnosis					
	IE, N = 18^1^	IT, N = 14^1^	ET, N = 29^1^	PV, N = 19^1^	Pre-PMF, N = 13^1^	Overt-PMF, N = 16^1^
**MF-0**	18 (100%)	14 (100%)	24 (83%)	13 (68%)	3 (23%)	
**MF-1**			5 (17%)	6 (32%)	10 (77%)	
**MF-2**						6 (37%)
**MF-3**						10 (63%)

### Comparison of clinical and laboratory characteristics between MPN and IE/IT patients

The characteristics with statistically significant differences between different groups (as showed in **Table 1**) were further investigated between clinical similar groups, comparisons were made between PV and IE (**Table 3**), and between ET and IT (**Table 4**). It is noteworthy that compared to their idiopathic mimickers, PV and ET patients both had significantly older age, higher platelet count, higher LDH and TNF-α concentration. The gender distribution of both PV and ET differs significantly from IE or IT. But IE patients are predominantly male (100%), while IT patients are predominantly female (93%), this phenomenon may be attributed to our small sample size. While the remaining characteristics showed no significant differences between ET and IT patients, PV patients also have higher WBC counts, higher MCV, higher basophil counts, higher IL-1β, higher IL-2R, lower EPO levels, and larger spleens compared to IE patients.

**Table 3:** Comparison of clinical and laboratory characteristics between PV and IE patients.

Characteristic	Diagnosis		p-value^2^
	PV, N = 19^1^	IE, N = 18^1^	
**Age, yrs**	59 (52, 68)	46 (29, 55)	0.003
**Gender**			<0.001
Male	5 (26%)	18 (100%)	
Female	14 (74%)	0 (0%)	
**WBC, ×10^9/L**	13.2 (8.4, 20.6)	7.1 (6.2, 7.8)	<0.001
**Hb, g/L**	178 (165, 194)	185 (182, 189)	0.289
**MCV, fL**	86 (83, 91)	93 (88, 95)	0.007
**PLT, ×10^9/L**	577 (419, 863)	193 (154, 242)	<0.001
**PDW, fL**	12.80 (11.65, 14.90)	13.10 (11.00, 14.05)	0.847
**P-LCR, %**	29 (24, 36)	31 (25, 34)	0.868
**Basophil, ×10^9/L**	0.05 (0.04, 0.08)	0.02 (0.02, 0.03)	<0.001
**LDH, U/L**	256 (219, 276)	187 (171, 225)	0.004
**EPO, pg/ml**	2 (2, 3)	10 (7, 15)	0.002
**IL-1b, pg/ml**	18 (6, 50)	5 (5, 9)	0.028
**IL-2R, pg/ml**	505 (292, 574)	327 (243, 351)	0.007
**TNF-a, pg/ml**	25 (18, 35)	7 (6, 12)	<0.001
**Size of spleen, cm**	4.10 (3.65, 4.60)	3.55 (3.20, 3.90)	0.010

^1^Median (IQR); n (%).

^2^Man-Whitney test; Pearson’s Chi-squared test.

**Table 4:** Comparison of clinical and laboratory characteristics between ET and IT patients.

Characteristic	Diagnosis		p-value^2^
	ET, N = 29^1^	IT, N = 14^1^	
**Age, yrs**	48 (36, 64)	27 (21, 45)	0.002
**Gender**			<0.001
Male	18 (62%)	1 (7%)	
Female	11 (38%)	13 (93%)	
**WBC, ×10^9/L**	8.4 (7.1, 10.4)	9.4 (7.2, 11.2)	0.873
**Hb, g/L**	133 (115, 143)	126 (116, 134)	0.288
**MCV, fL**	89 (87, 92)	85 (83, 86)	0.200
**PLT, ×10^9/L**	821 (692, 1,099)	528 (474, 615)	0.024
**PDW, fL**	11.20 (9.80, 12.60)	10.15 (9.55, 11.98)	0.578
**P-LCR, %**	24 (19, 30)	20 (18, 28)	0.487
**Basophil, ×10^9/L**	0.05 (0.02, 0.11)	0.02 (0.01, 0.05)	0.338
**LDH, U/L**	231 (206, 271)	179 (146, 194)	0.004
**EPO, pg/ml**	6 (4, 18)	8 (7, 12)	0.223
**IL-1b, pg/ml**	5 (5, 5)	5 (5, 5)	0.932
**IL-2R, pg/ml**	456 (308, 562)	349 (267, 424)	0.113
**TNF-a, pg/ml**	19 (13, 30)	7 (6, 11)	0.021
**Size of spleen, cm**	4.10 (3.50, 4.70)	3.50 (3.20, 3.80)	0.106

^1^Median (IQR); n (%).

^2^Man-Whitney test; Pearson’s Chi-squared test.

**Figure 4:**
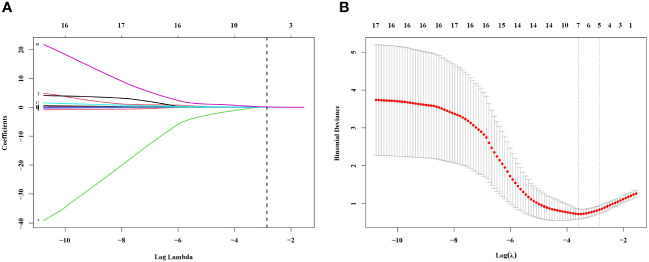
Feature Selection Using the LASSO Binary Logistic Regression Model. **(A)** Log (Lambda) value of the 18 features in the LASSO model. A coefficient profile plot was produced against the log (lambda) sequence. **(B)** Parameter selection in the LASSO model used tenfold cross-validation via minimum criterion. Partial likelihood deviation (binomial deviation) curves and logarithmic (lambda) curves were plotted. Use the minimum standard and 1se (1-*SE* standard) of the minimum standard to draw a vertical dashed line at the optimal value. The optimal lambda produced four nonzero coefficients. LASSO, least absolute shrinkage and selection operator; *SE*, standard error.

These findings provide valuable insights into the baseline differences between the MPN and IE/IT groups, suggested that these two conditions, MPN and IE/IT, can be distinguished through clinical and laboratory parameters. In order to construct a diagnostic model for distinguishing between all subtypes of MPN and IE/IT, we included the 18 clinical and laboratory variables collected from all patients into LASSO logistic regression analysis to analyze their ability to differentiate between MPN and IE/IT. Five variables were selected based on non-zero coefficients calculated by LASSO (**Figures 4A, B**). These selected features included age, Hb level, PLT count, serum TNF-α level, size of spleen. These features were subsequently included in multivariate logistic regression analysis.

### Development of an individualized MPN prediction model

**Figure 5:**
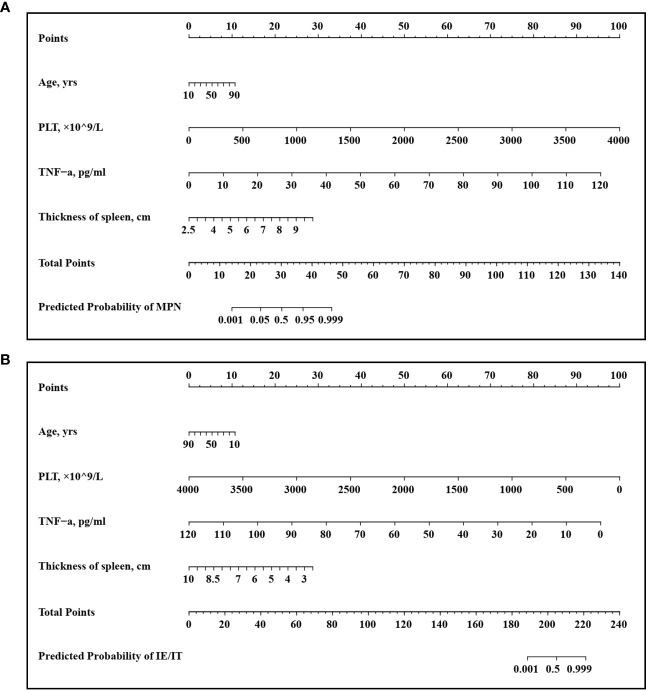
Developed Nomogram. This nomogram was developed with age, PLT count, TNF-α concentration, spleen size. **(A)** Nomogram for predicting MPN in newly onset erythrocytosis or thrombocytosis patients; **(B)** Nomogram for predicting IE/IT in newly onset erythrocytosis or thrombocytosis patients.

A multivariate logistic regression analysis identified age (*OR:* 1.06 [95% CI: 1.00–1.13] p=0.048), PLT count (*OR:* 1.01 [95% CI: 1.01–1.02] p=0.001), serum TNF-α level (*OR:* 1.40 [95% CI: 1.15–1.95] p=0.014), size of spleen (*OR:* 5.06 [95% CI: 1.49–26.25] p=0.023) were independent predictive factors available for differentiating newly onset MPN and IE/IT (**Table 5**). The above independent predictors were then incorporated to develop predictive nomograms to predict probabilities of MPN and IE/IT (**Figure 5**).

**Table 5:** Prediction factors for MPN and IE/IT in newly onset erythrocytosis and thrombocytosis patients.

Intercept and variable	Coefficient	Odds ratio (95% CI)	p-value
Intercept	-17.759		
Age, yrs	0.057	1.06 (1.00-1.13)	0.048
Hb, g/L	0.002	1.00 (0.98-1.03)	0.904
PLT, ×10^9^/L	0.011	1.01 (1.01-1.02)	0.001
TNF-α, pg/ml	0.336	1.40 (1.15-1.95)	0.014
Thickness of spleen, cm	1.622	5.06 (1.49-26.25)	0.023

### Performance and validation of the nomogram

**Figure 6:**
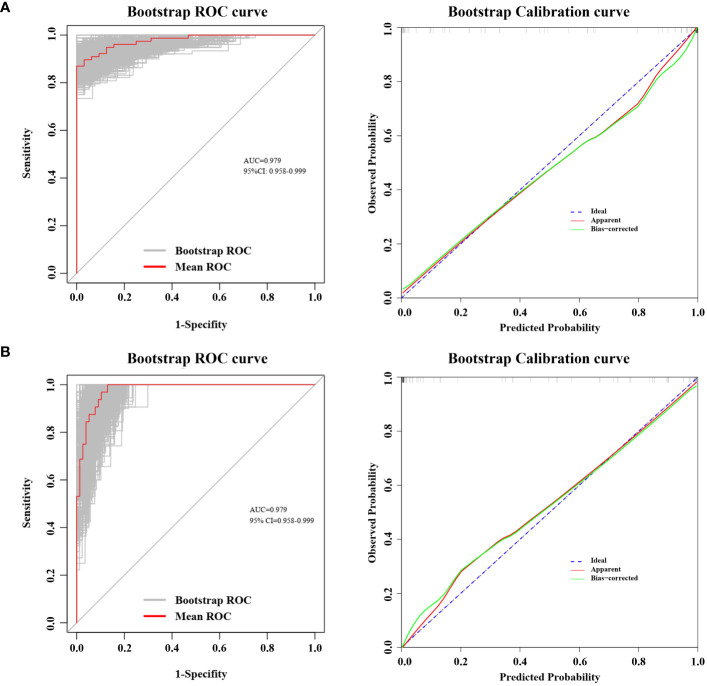
ROC Curve and Calibration Curve of Nomogram Model. Both ROC and calibration curve was performed via 1000 bootstrap iterations. ROC curve, The 45-degree diagonal line represented the baseline for random classification. The red line represented the mean ROC of 1000 bootstrap iterations. The grey lines represented the actual ROC of 1000 bootstrap iterations. (CI, confidence interval.) Calibration curve, The 45-degree diagonal line represented the ideal performance, where actual observed values perfectly match the model-predicted values. The apparent line refers to the line obtained by the observed model calibration curve. The bias-corrected line refers to the line obtained after making adjustments to the observed model calibration curve to correct its bias. **(A)** ROC curve and Calibration curve for predicting MPN in newly onset erythrocytosis or thrombocytosis patients; **(B)** ROC curve and Calibration curve for predicting IE/IT in newly onset erythrocytosis or thrombocytosis patients.

In our cohort, the ROC curve of the TNF-α incorporated nomogram model for MPN and IE/IT diagnosis showed good diagnostic efficacy (**Figure 6A**). The C-index for the prediction of TNF-α incorporated nomogram model was 0.978 (95% CI: 0.958-0.999) and was confirmed to be 0.979 (95% CI: 0.958-0.999) via 1000 bootstrap iterations validation. At the optimal cutoff value, the sensitivity for predicting MPN in newly onset patients with erythrocytosis or thrombocytosis was 85.7%, with a specificity of 100%; correspondingly, the sensitivity for predicting IE/IT in newly onset patients with erythrocytosis or thrombocytosis was 100%, with a specificity of 85.7%. The bootstrapped calibration curve closely follows the 45-degree diagonal line, indicating a strong agreement between predicted and observed probabilities (**Figure 6B**). In addition, the p-value of Hosmer–Lemeshow test is 0.998, suggested that the model was of goodness-of-fit.

## Discussion

To our knowledge, this is the first study to utilize cytokines as biomarkers for distinguishing between MPN and IE/IT in diagnostic procedures. In this retrospective study, we noticed that serum TNF-α level was significantly higher in MPN patients than those with IE/IT. Moreover, there is a correlation between TNF-α and grade of myelofibrosis, with patients having higher grades of MF associated with serum higher TNF-α. Integrated TNF-α with age, PLT count and spleen size into nomograms has demonstrated a rapid and convenient preliminary screening tool to assist physicians in efficiently distinguish MPN patients and IE/IT patients among newly onset erythrocytosis or thrombocytosis patients.

The inclusion of cytokine profiles contributes valuable insights into the underlying biological processes associated with MPN. Cytokines like TNF-α, being key signaling molecules, reflect the intricate interplay within the hematopoietic system. The excessive TNF-α cause various profound effect on the milieu of MPN: platelet hyperreactivity and mitochondrial dysfunction (17); venous thromboembolism risk (18); accelerate fibrocyte production and reticulin fibrosis deposition in ASXL1-mutated patients (19); favors the JAK2V617F mutated cells in survival and clonal expansion over unmutated cells (20, 21). Treatment approaches targeting TNF-α signaling are also under investigation, αTNFR1 and αTNFR2 antibody treatment demonstrated a certain level of therapeutic efficacy in a murine model of MPN (22). And etanercept, a soluble TNF-α receptor, improved constitutional symptoms in 60% participants and cytopenia or spleen size in 20% participants of a pilot clinical trial of 22 myelofibrosis patients (23). The primary source of excessive TNF-α remained elusive, various types of cell could produce TNF-α, including mutated and unmutated monocytes, megakaryocytes and platelets, hematopoietic stem cells lymphocytes and stromal cells, it is noteworthy that recent study indicate that MDSC may also participate in the secretion of TNF-α (11, 15, 20, 24, 25).

At the optimal cutoff value, the sensitivity for predicting IE/IT or the specificity for predicting MPN can reach 100%; while in the same setting, the specificity for predicting IE/IT or the sensitivity for predicting MPN is 85.7%. This indicates that our model can accurately identify IE/IT patients, but may misclassify a small proportion of atypical cases of MPN patients (referring to patient with young age, low platelet count, and low TNF levels, with small spleen) as IE/IT.

The accurate identification of MPN patients is crucial for timely and appropriate interventions, as treatments are different for different subtypes of MPN and IE/IT: Firstly, for ET and PV patients, cardiovascular risk factors need to be managed, and aspirin is recommended except for contraindications. Cytoreductive drugs, such as hydroxyurea, pegylated IFN-γ and ruxolitinib, should be used for both low-risk and high-risk patients who meet the indications for cytoreductive therapy. Allogeneic bone marrow transplantation is currently the only treatment that can cure PMF, but treatment-related mortality is high. Ruxolitinib is a targeted therapy for the JAK/STAT pathway in the pathogenesis of PMF (26, 27). while IE/IT patients should first exclude the possible underlying primary disease, as hypoxic pulmonary diseases, cardiac and renal disorders, tumors are common causes for secondary erythrocytosis; anemia, infection and inflammation usually cause secondary thrombocytosis (28, 29). Traditionally, in the differential diagnosis of IE/IT and MPN, a bone marrow biopsy must be performed after excluding possibilities of peripheral cytosis secondary to underlying primary diseases (1). Our nomograms utilize only routine blood tests, cytokine and ultrasonic data makes it a feasible and accessible tool for clinical practice. Its simplicity and accuracy position it as a valuable decision support tool for healthcare professionals in different settings.

While these findings underscore the promising nature of using cytokines as biomarkers to assist the diagnosis of MPN and IE/IT, it is essential to acknowledge potential limitations. This model cannot replace the bone marrow biopsy, which is the gold standard in bone marrow-related hematologic disorders. And this model may potentially miss the diagnosis of a small portion of ET patients. Further prospective cohort studies and external validation are still needed to identify more accurate and appropriate diagnostic biomarkers. Additionally, the underlying mechanism of elevation of TNF-α and its impact on the pathogenesis of MPN are still in need of exploration.

## Conclusion

In conclusion, the elevation of serum TNF-α in MPN patients is of diagnostic significance and is correlated with the severity of myelofibrosis. The nomogram incorporating TNF-α with age, PLT count and spleen size presents a noteworthy tool in the preliminary discrimination of MPN patients and those with idiopathic erythrocytosis or thrombocytosis. This highlights the potential of cytokines as biomarkers in hematologic disorders.

## Data availability statement

The raw data supporting the conclusions of this article will be made available by the authors, without undue reservation.

## Ethics statement

The study had approval from the institutional review board of Tongji Hospital, Tongji Medical College, Huazhong University of Science and Technology. All procedures followed were in accordance with the ethical standards of the responsible committee on human experimentation (institutional and national) and with the Helsinki Declaration. Informed consent was obtained from all patients for being included in the study.

## Author contributions

ZW: Data curation, Formal analysis, Investigation, Methodology, Visualization, Writing – original draft. YM: Data curation, Formal analysis, Investigation, Writing – original draft. ZY: Data curation, Investigation, Writing – original draft. QG: Software, Visualization, Writing – original draft. HX: Data curation, Funding acquisition, Project administration, Writing – review & editing. ZH: Formal analysis, Funding acquisition, Project administration, Supervision, Writing – review & editing. ZH: Conceptualization, Funding acquisition, Project administration, Resources, Supervision, Writing – review & editing.
